# Design guide for synthetic small regulatory RNAs for high-efficiency gene knockdown in bacteria

**DOI:** 10.71150/jm.2603026

**Published:** 2026-07-06

**Authors:** Jun Ren, Yubin Kim, Hyun Jung Nam, Hyang-Mi Lee, Dokyun Na

**Affiliations:** Department of Biomedical Engineering, Chung-Ang University, Seoul 06974, Republic of Korea

**Keywords:** synthetic sRNA, post-transcriptional regulation, synthetic biology, metabolic engineering, gene expression, knockdown

## Abstract

Small regulatory RNAs (sRNAs) are short noncoding RNAs that can fine-control the expression of target genes in trans at the post-transcriptional level in prokaryotes. Since there is a big challenge in constructing gene-knockout libraries, synthetic sRNAs have attracted considerable interest in synthetic biology and metabolic engineering, as they enable targeted gene knockdown without requiring chromosomal modifications. However, the development of high-efficiency synthetic sRNAs remains a demanding task that requires careful consideration of multiple design factors. Here, we provide a detailed protocol for the design and construction of synthetic sRNAs, detailing key design principles and critical optimization factors, including scaffold selection, target mRNA binding affinity, target mRNA secondary structure, and Hfq expression levels. This strategy can be broadly applied across *E. coli* and other bacterial hosts to modulate gene expression, thereby supporting versatile applications in synthetic biology and metabolic engineering.

## Overview

Small noncoding RNAs were first identified as regulators of bacterial gene expression in 1961 (Jacob and Monod, 1961), and have since been recognized as a widely conserved mechanism across diverse bacterial species (Storz et al., 2005). Among these, small regulatory RNAs (sRNAs) constitute a major class of trans-acting molecules that modulate mRNA translation and stability at the post-transcriptional level.

In recent years, trans-acting repression systems such as CRISPR interference (CRISPRi) have become powerful tools for targeted gene knockdown (Wang et al., 2018). CRISPRi enables rapid transcriptional repression without requiring chromosomal modification, and platforms such as Mobile-CRISPRi have expanded its applicability to non-model bacteria (Peters et al., 2019). However, the practical deployment of CRISPR-based systems can be constrained by the metabolic load associated with expressing catalytically dead Cas9 (dCas9), which is known to reduce cellular fitness and introduce stress responses (Vento et al., 2019).

Synthetic sRNA provides a low-burden and flexible alternative to CRISPRi for both functional studies and metabolic engineering (Cho et al., 2023; Georg et al., 2025; Na et al., 2013; Ren et al., 2024). Because sRNAs function as short RNA regulators rather than large protein effectors, their expression imposes minimal translational and metabolic costs on the host cell. Due to their non-integrative episomal delivery, which bypasses genomic modification, these systems facilitate rapid and reversible knockdown of target genes. Furthermore, their binding affinity to target mRNAs can be precisely modulated by optimizing sequence complementarity, thereby enabling fine-tuned control over gene silencing efficiency (Na et al., 2013; Ren et al., 2024). These features make synthetic sRNAs particularly advantageous for probing gene function and evaluating metabolic phenotypes, including product titers, growth rate, and cellular physiology (Cho et al., 2023; Na et al., 2013; Yeom et al., 2022). Given the broad conservation of trans-acting sRNA mechanisms across bacterial species, synthetic sRNAs engineered for a specific host can be readily adapted to others (Cho et al., 2023; Yeom et al., 2022). Consequently, synthetic sRNA-based platforms have emerged as versatile and scalable tools for synthetic biology and metabolic engineering across diverse bacterial hosts.

To enhance the utility of synthetic sRNA as a practical tool, we recently developed a workflow to establish design principles for synthetic sRNAs in *Escherichia coli* (Fig. 1), which can be transferable to other bacterial species, and derived four design rules that extend beyond previously established rules in *E. coli* (Na et al., 2013; Ren et al., 2024; Yoo et al., 2013). In the workflow, **(1) Scaffold selection:** we screened about 150 *E. coli* native sRNAs based on three criteria to identify sRNA scaffolds that outperform the MicC-based system previously shown to exhibit high repression efficiency (Na et al., 2013): long in vivo half-life, experimentally validated target-binding boundaries, and AU-rich Hfq-binding motifs. **(2) Effective target-binding affinity determination:** To evaluate scaffold-specific performance, diverse synthetic sRNAs targeting three fluorescent mRNAs (DsRed2, tdTomato, and mCherry) were designed with consistent binding affinities (approximately -20 kcal/mol) and scaffold types, and their repression efficiencies were systematically assessed. **(3) Translation initiation region (TIR) accessibility determination:** The secondary structure of the mRNA TIR significantly affects ribosome accessibility and, consequently, translational efficiency. Therefore, the hybridization of synthetic sRNAs with their target mRNAs is inherently influenced by the pre-existing TIR structure. Through a systematic 5'-UTR mutational analysis using a library of 100 TIR variants, we evaluated the repression efficiencies of synthetic sRNAs and established the thermodynamic threshold required for stable and predictable gene silencing. **(4) Hfq level optimization:** The RNA chaperone Hfq is indispensable for facilitating synthetic sRNA-mRNA pairing and subsequent target mRNA degradation. Since endogenous Hfq levels are typically optimized for native sRNA pools, the introduction of high-level synthetic sRNAs may exceed the host’s Hfq capacity. To supplement Hfq without perturbing its global regulatory functions, we engineered an *hfq* expression system with modified ribosome-binding sites (RBS), identifying an optimal Hfq level that maximizes repression potency while maintaining cellular fitness.

Through the workflow, we derived four fundamental design principles for achieving high-efficiency gene knockdown in *E. coli*. **(1) Scaffold selection rule:** the SgrS-derived scaffold was identified as the most efficient and stable platform in *E. coli*, consistently providing robust and consistent repression across diverse target mRNAs. **(2) Target-binding affinity rule:** the hybridization free energy between the synthetic sRNA and its target mRNA should be optimized within the range of -20 to -40 kcal/mol for effective silencing. Notably, a binding energy of approximately -30 kcal/mol is sufficient to achieve a repression efficiency exceeding 90%. **(3) TIR accessibility rule:** the accessibility of the TIR is a critical determinant, where the Gibbs free energy of the mRNA secondary structure should be higher than -16 kcal/mol to ensure sufficient sRNA-mRNA pairing. If the TIR forms a more stable structure (with a free energy lower than -16 kcal/mol), the repression efficiency is significantly diminished due to restricted accessibility. **(4) Hfq level optimization rule:** in the presence of the native chromosomal *hfq* gene, supplementing the host with an additional 0.2-fold of the wild-type *hfq* expression level markedly enhances the efficacy of the synthetic sRNA regulatory system.

## Applications

Synthetic sRNAs have been widely adopted in metabolic engineering to modulate gene expression, as precise and tunable control of gene expression is essential for enhancing the production of high-value metabolites (Cho et al., 2023; Lins et al., 2021; Sittka et al., 2008; Yeom et al., 2022; Zhang et al., 2003). Accordingly, synthetic sRNA-based regulation has been successfully applied to enhance the biosynthesis of diverse compounds, including cadaverine, tyrosine, proline, putrescine, and violacein in *E. coli*, as well as valerolactam and methyl anthranilate in *Corynebacterium glutamicum* (Cho et al., 2023; Na et al., 2013; Noh et al., 2017; Yeom et al., 2022).

Beyond simple repression, synthetic sRNAs can also enhance the stability of complex synthetic genetic circuits. Genetic circuits are often vulnerable to noise-induced disruption of programmed behaviors, as transcriptional leakiness generates unintended basal transcripts that spuriously activate regulatory pathways (Eldar and Elowitz, 2010; Huang et al., 2015). This intrinsic sensitivity to stochastic fluctuations poses a major challenge to reliable circuit implementation. To overcome this limitation, synthetic sRNAs have recently been incorporated as specialized leak suppressors that preferentially eliminate unintended basal transcripts (Ren et al., 2024; Tran et al., 2025) (Fig. 2).

Recent studies have demonstrated the effectiveness of this strategy. Jun and colleagues showed that optimized synthetic sRNAs effectively suppressed basal leakage of tdTomato expression driven by both T7 and *lac* promoters across a broad range of IPTG concentrations, including non-induced conditions (Fig. 2A). Moreover, when the optimal synthetic sRNAs simultaneously targeted both inducers within an artificial positive-feedback circuit, expression noise was significantly reduced (Ren et al., 2024) (Fig. 2B). Similarly, Tran and coworkers implemented synthetic sRNAs in an ‘expiry-date’ genetic circuit composed of different sequential transcriptional activation steps mediated by diverse transcription factors (TFs) that culminate in expression of Lysis E (Tran et al., 2025) (Fig. 2C). Because unintended basal expression of Lysis E disrupts programmed circuit timing, a synthetic sRNA was designed to specifically target its mRNA. Incorporation of this synthetic sRNA markedly decreased leakiness, restored normal cellular growth under permissive conditions, and preserved the intended temporal control of the genetic ‘expiry-date’ circuit.

Taken together, these examples highlight synthetic sRNAs as versatile tools for both metabolic engineering and synthetic biology, enabling precise gene regulation and robust circuit behavior. In the following section, we provide detailed, step-by-step protocols for the rational design of synthetic sRNAs to achieve predictable and efficient gene expression control.

## Methods

### Plasmids for synthetic sRNA expression and Hfq expression

The plasmid maps for synthetic sRNA expression and the genetic sequences of *hfq* gene and synthetic sRNA gene are shown in Figs. S1 and S2. The whole plasmid sequences in SnapGene format are provided as Data S1 and S2.

#### 1. Plasmid for expressing synthetic sRNA (pWAS2)

The plasmid for constitutive synthetic sRNA expression (pWAS2, Addgene #251406) was derived from the pWAS vector, which was previously utilized to express synthetic sRNAs harboring the MicC scaffold (Na et al., 2013) (Fig. S2B). The plasmid was modified to harbor the SgrS scaffold under the control of the phage λ P_R_ promoter (BBa_R0051) and the T1/TE terminator (BBa_B0025). The native *ptsG*-targeting sequence was removed from the wild-type SgrS. To construct functional synthetic sRNAs, a newly designed target-binding sequence must be inserted at the site previously occupied by the *ptsG*-targeting sequence (Fig. S2).

When tighter or inducible control of synthetic sRNA expression is required, particularly in cases where strong repression may impair cell growth or viability, an additional regulatory module can be employed. In this system, the optimal strength of phage λ P_R_ promoter is regulated by the temperature-sensitive repressor CI^ts2^ expressed by the P_BAD_ promoter. Because the plasmid also constitutively express AraC, synthetic sRNA transcription becomes dependent on arabinose induction. This configuration enables stringent and conditional control of synthetic sRNA expression (Sung et al., 2016). Specifically, at 25°C in the presence of arabinose, CI^ts2^ is produced and remains active, thereby repressing the P_R_ promoter and preventing synthetic sRNA production. In contrast, at 37°C in the absence of arabinose, CI^ts2^ becomes inactive, allowing P_R_ promoter to produce synthetic sRNAs.

#### 2. Plasmid for supplementing Hfq protein (pSC101-*hfq2*)

Hfq protein plays an essential role in the suppression of mRNA translation by synthetic sRNA (Moller et al., 2002; Zhang et al., 2002). Hfq facilitates the hybridization of synthetic sRNA and its target mRNA, and also facilitates the recruitment of RNase E for rapid degradation of target mRNA (Morita and Aiba, 2011). As synthetic sRNA is produced at a large quantity from the strong promoter, the native level of Hfq is insufficient for the facilitated translation suppression by synthetic sRNA (Ren et al., 2024) and, therefore, Hfq protein should be additionally produced. To supply Hfq, the *hfq* gene along with its native promoter was PCR-amplified from *E. coli* DH5α genome and cloned into the pSC101 plasmid (low copy number) (Ren et al., 2024). As Hfq is an RNA chaperone and global regulator and its uncontrolled expression may distort cellular physiology, we used its native promoter for the additional *hfq* gene to be controlled naturally. For optimizing the level of Hfq, we engineered the ribosome-binding site (RBS) of the *hfq* gene using RBSDesigner, resulting in diverse translation efficiencies (Na and Lee, 2010; Ren et al., 2024; Vo et al., 2021). One of the *hfq* variants (approximately 0.2-fold that of the wild-type *hfq*) produced the highest repression efficiency (pSC101-*hfq2*, Addgene #251407, Fig. S1A). Co-expression of the *hfq* variant with synthetic sRNA resulted in up to 90% reduction in DsRed2, mCherry, and tdTomato expression. Since the optimal Hfq level is dependent on the synthetic sRNA amount, the *hfq* gene should be further re-optimized if synthetic sRNA is driven by promoters of different strengths.

### Plasmid for target gene expression

Synthetic sRNAs serve as versatile tools for repressing both endogenous chromosomal genes in metabolic engineering and exogenous targets within plasmid-borne genetic circuits in synthetic biology. While their primary application often involves the modulation of host metabolism, this protocol utilizes standardized reporter plasmids expressing fluorescent proteins to facilitate the quantitative and direct evaluation of synthetic sRNA-mediated repression performance. Specifically, three fluorescent genes—*DsRed2* (Na et al., 2013), *mCherry* (Addgene #29769), and *tdTomato* (Addgene #44640) are employed as representative targets under the transcriptional control of the *lac* promoter. These reporter constructs are maintained on a p15A replication origin (medium copy number) and harbor a chloramphenicol resistance marker along with T1/TE transcriptional terminators to ensure stable expression and insulation. The specific mRNA sequences within each fluorescent reporter targeted by the synthetic sRNAs are detailed in Fig. 3.

### Workflow for establishing design rules for synthetic sRNAs

#### 1. Selection of sRNA scaffold

To ensure robust gene knockdown, selecting an appropriate sRNA scaffold is a prerequisite, as the scaffold's intrinsic stability and its affinity for the Hfq chaperone significantly dictate the overall repression efficiency (Morita and Aiba, 2011; Zhang et al., 2002). In a previous study, a systematic screening of endogenous *E. coli* sRNAs was conducted to identify high-performance scaffolds (Na et al., 2013; Ren et al., 2024). The selection was based on three key functional determinants: a long in-vivo half-life (> 30 min) to ensure stability, well-defined target-binding boundaries to minimize off-target interference, and the presence of AU-rich motifs for efficient Hfq recruitment. Among the evaluated candidates, the SgrS-derived scaffold demonstrated the most consistent and superior repression performance across various mRNA contexts in *E. coli*. Consequently, we recommend utilizing the SgrS scaffold as the default choice for high-efficiency synthetic sRNA design in *E. coli*.

#### 2. Hybridization energy between synthetic sRNA and its target mRNA

The mRNA region targeted by synthetic sRNA is typically selected to span from immediately after the AUG start codon to approximately 20 nucleotides (nt) downstream within the coding sequence (CDS) (Fig. 3). This specific positioning ensures robust translational repression while minimizing potential off-target effects (Na et al., 2013). The reverse complementary sequence of the identified mRNA target region is employed as the target-binding sequence within the synthetic sRNA. This complementarity facilitates stable hybridization, thereby effectively inhibiting translation of the target mRNA.

The repression efficiency of synthetic sRNAs is dependent on the hybridization energy between the target-binding sequence of the synthetic sRNA and the target region of the mRNA (Na et al., 2013). Synthetic sRNAs with similar binding affinities exhibit comparable repression efficiencies, even when specific nucleotide mutations are introduced to modulate the free energy (Na et al., 2013; Ren et al., 2024). Consequently, binding energy is the key design parameter for achieving the desired repression level.

The hybridization energy between a synthetic sRNA and its target mRNA should be sufficiently strong (more negative than -20 kcal/mol) to ensure stable repression, but sufficiently weaker (more positive than -40 kcal/mol) to avoid cross-repression (Na et al., 2013; Ren et al., 2024). This hybridization energy can be calculated using RNAup (Mückstein et al., 2006).

#### 3. Secondary structure of translation initiation region within target mRNA

During translation initiation, the secondary structure surrounding the TIR—encompassing the SD sequence and its downstream regions—influences ribosome binding (Unoson and Wagner, 2007; Vimberg et al., 2007). Specifically, a stable helical structure within or near the TIR can sterically hinder ribosome access, thereby reducing translational efficiency (Unoson and Wagner, 2007). Likewise, stable secondary structures formed around the synthetic sRNA target regions on an mRNA can interfere with synthetic sRNA binding, as the energetic cost required to unfold these pre-existing structures may limit efficient synthetic sRNA-mRNA base pairing.

The impact of the TIR secondary structure on synthetic sRNA efficiency was previously established through an extensive UTR library analysis (Ren et al., 2024). In that study, distinct tdTomato UTR variants were generated by introducing random mutations into the 5′-UTR to evaluate synthetic sRNA-mediated repression. Structural analysis revealed that TIR accessibility is a critical determinant of repression efficiency of synthetic sRNAs, with robust translational repression most consistently achieved when the structural free energy of the TIR is higher than −16 kcal/mol. Consequently, the likelihood of successful repression can be predicted by calculating the Gibbs free energy of the mRNA secondary structure.

#### 4. Hfq expression level optimization

Hfq, a well-characterized RNA chaperone, plays a central role in sRNA-mediated post-transcriptional regulation by facilitating sRNA–mRNA hybridization (Moller et al., 2002; Zhang et al., 2002), protecting sRNAs from degradation (Schu et al., 2015), and promoting target mRNA decay (Morita and Aiba, 2011). To enhance synthetic sRNA function, Hfq expression level should be optimized to accommodate the increased regulatory load (Ren et al., 2024; Vo et al., 2021). Among the RBS variants evaluated, an *hfq* variant expressed at approximately 0.2-fold the level of the wild-type *hfq* produced the most robust repression (Ren et al., 2024).

It is important to note, however, that this optimized Hfq level was specifically determined for synthetic sRNAs using the SgrS-derived scaffold and produced by a strong promoter. Therefore, the Hfq level may require further re-optimization if the synthetic sRNA abundance, the choice of scaffold, or the host species is varied. Such fine-tuning of Hfq expression can be achieved by engineering the RBS using various computational design tools (Na and Lee, 2010; Salis, 2011; Seo et al., 2013, 2014).

#### 5. Design guidelines for extending synthetic sRNA-based silencing to diverse bacterial hosts

To extend the application of synthetic sRNAs to diverse non-model bacterial hosts, several factors must be considered. Notably, a significant portion of the design parameters for synthetic sRNAs are engineered to be host-independent. First, the modular architecture of the synthetic sRNA—which separates the scaffold from the target-binding sequence—ensures that the silencing mechanism remains largely independent of the host-specific genetic context. Furthermore, the accessibility of the target mRNA is primarily governed by the intramolecular interactions of ribonucleotides and the thermodynamic stability of the sRNA-mRNA complex, both of which are features inherently independent of the bacterial host.

Despite these host-orthogonal features, a primary consideration for successful implementation in non-model hosts is the selection of an optimal scaffold that can effectively interact with the host’s endogenous Hfq chaperone. This is necessary because endogenous Hfq may fail to stabilize the synthetic sRNA-mRNA complex due to variations in binding motifs or Hfq abundance across different species. Such a scaffold can be identified through bioinformatic and experimental screening of the native sRNA repertoire within the target host. However, recent studies have demonstrated strategies to circumvent the requirement for host-specific scaffolds. For instance, synthetic sRNAs utilizing the RoxS scaffold, supplemented with the heterologous expression of the Hfq chaperone from *Bacillus subtilis*, have shown efficient gene knockdown across a wide range of bacterial taxa (Cho et al., 2023). Similarly, the MicC scaffold enabled significant gene repression in *Corynebacterium glutamicum* when coupled with the co-expression of the *E. coli* Hfq chaperone (Sun et al., 2019). Interestingly, certain scaffolds like RyhB can facilitate effective gene silencing in *Methylomonas* sp. DH-1 even without exogenous Hfq supplementation (Ren et al., 2020). Consequently, if the identification of a host-specific scaffold proves challenging, we recommend initially testing designed synthetic sRNAs without auxiliary chaperones. If silencing efficiency is insufficient, the co-expression of the Hfq protein from the target host, or a well-characterized homolog from other species, should be implemented to ensure robust gene knockdown.

## Materials

All solutions and buffers are prepared under sterile conditions and stored according to the manufacturer’s instructions. Plasmids, enzymes, competent cells, antibiotics, and other consumables used in this protocol are listed in the corresponding sections. Fluorescence measurements are performed using appropriate excitation and emission filter sets, as detailed in the Protocols section.

### A. Chemicals and reagents

- DNA ladder, 1 kb plus (Cat#SM354-500; Biofact)

- DNA ladder, 100 bp (Cat#SM342-500; Biofact)

- Ampicillin (Cat#A9518; Sigma-Aldrich)

- Bacto tryptone (Cat#211705; BD Biosciences)

- Yeast extract (Cat#212750; BD Biosciences)

- Bacto agar (Cat#214010; BD Biosciences)

- Sodium chloride (Cat#13-1050-01; Junsei)

- H_2_O (sterile)

- Ecodye^TM^ nucleic acid stain (Cat#ES301-1000; Biofact)

- Tris-acetate-EDTA buffer (Cat#T6025; Sigma-Aldrich)

- Higel agarose (Cat#HA0100500; Bio electronics company)

- L-arabinose (Cat#A3256; Sigma-Aldrich)

- DNase/RNase-free water (Cat#W4502; Sigma-Aldrich)

- Glycerol (Cat#G9012; Sigma-Aldrich)

### B. Bacteria, plasmids, and culture media

- *E. coli* strain DH5α (Cat#18265-017; Invitrogen)

- pWAS2 plasmid for synthetic sRNA expression (Addgene #251406, Fig. 4A).

- Plasmids constructed for the expression of fluorescent reporter genes-*DsRed2* (Na et al., 2013), *mCherry* (Addgene #29769), and *tdTomato* (Addgene #44640).

- pSC101-*hfq2* plasmid (Addgene #251407), harboring the *hfq* variant (*hfq*), including the native *hfq* promoter, an engineered RBS, the *hfq* coding sequence, and a transcription terminator (Figs. 4B and S1B).

- Luria-Bertani (LB) medium: LB medium is prepared by dissolving 10 g of Bacto tryptone, 5 g of yeast extract, and 10 g of NaCl in 1 L of distilled water. For the preparation of solid medium, the liquid LB medium is supplemented with 15 g of Bacto agar per liter. All media are sterilized via autoclaving at 121°C for 15 min.

- Liquid and solid LB media are supplemented with antibiotics as required. For strains harboring pWAS2, ampicillin is added to a final concentration of 100 μg/ml; for reporter gene plasmids, chloramphenicol is added to 25 μg/ml; and for pSC101-*hfq2*, spectinomycin is added to 50 μg/ml.

### C. Equipment and kits

- PCR instrument (Cat#1861096; Bio-Rad)

- Tabletop microcentrifuge (Cat#5702R; Eppendorf AG)

- Nucleic acid electrophoresis equipment (Cat#AD110; Takara)

- Gel imaging system (Cat#170-8195; Bio-Rad)

- Vortex mixer (Cat#G560E; Scientific Industries Inc)

- Water bath (Cat#TSGP02; Thermo Fisher Scientific)

- Sterile 34 ml culture tube (Cat#9826; Corning)

- Sterile 250 ml culture flask (Cat#4442-250; Corning)

- Multi-well plates (Cat#3595/3548; Corning)

- 0.5 ml PCR reaction tubes (Cat#TWI-0201/TBI-0501; Bio-Rad)

- Nanodrop ND-1000 spectrophotometer (Cat#ND-2000; Thermo Fisher Scientific)

- Universal gel & PCR clean-up kit (Cat#BNROP-0067; Bionics)

- Plasmid DNA miniprep S & V kit (Cat#BNROP-0068; Bionics)

- Guava EasyCyte flow cytometer (Millipore, Germany)

### D. Primers and enzymes

- Primers used for anti-DsRed2 synthetic sRNA construction and validation are listed in Table 1.

- Restriction enzymes: EcoRI (Cat#R0101; NEB), XhoI (Cat#R0146; NEB), KpnI (Cat#R0142; NEB), SacI (Cat#R0156S; NEB), PacI (Cat#R0547S; NEB), DpnI (Cat#R0176; NEB)

- Q5 High-Fidelity DNA Polymerase (Cat#M0491S; NEB)

- T4 polynucleotide kinase (Cat#M0201S; NEB)

- T4 DNA ligase (Cat#M0202S; NEB)

- *Taq* DNA polymerase (Cat#ST116-25h; Biofact)

### E. Bioinformatics tools for target binding sequence design

- SnapGene to visualize plasmid maps and simulate cloning procedures: https://www.snapgene.com/

- RNAup to predict the hybridization free energy between the target-binding sequence of the synthetic sRNA and its corresponding target region on the mRNA: http://rna.tbi.univie.ac.at/cgi-bin/RNAWebSuite/RNAup.cgi (Mückstein et al., 2006).

- RNAfold to calculate the Gibbs free energy of the secondary structure within the mRNA, ensuring that the TIR remains structurally accessible for synthetic sRNA binding: http://rna.tbi.univie.ac.at/cgi-bin/RNAWebSuite/RNAfold.cgi (Mathews et al., 2004).

## Protocols

This protocol describes the stepwise design, construction, and expression of synthetic sRNAs, including target-binding sequence design, plasmid construction by inverse PCR, template removal and ligation, transformation into *E. coli*, colony screening, and sequence verification. DNA sequencing was performed by a commercial service provider and is therefore not described in detail.

### A. Design of target-binding sequences

1. *Target gene selection:* Select a target gene and verify its coding sequence using standard databases, such as EcoCyc, http://ecocyc.org; GenBank, http://www.ncbi.nlm.nih.gov/genbank/; EMBL, http://www.ebi.ac.uk; and KEGG, http://www.kegg.jp/kegg/genes.html

2. *Target region identification:* Design a 20-nt target-binding sequence complementary to the N-terminal coding region immediately downstream of the start codon. Targeting this region efficiently blocks ribosome binding and minimizes off-target effects relative to 5’-UTR targeting (Na et al., 2013; Ren et al., 2024) (Fig. 3).

3. *Example implementation:* The fluorescent reporter DsRed2 was used as an example target to illustrate the design process (Fig. S2A). For instance, when the selected mRNA target sequence is 5’-GCGAGCAGUGAGAACGUCAU-3’, the corresponding complementary target-binding sequence for the synthetic sRNA is 5’-AUGACGUUCU CACUGCUCGC-3’.

4. *Binding energy between synthetic sRNA and its target mRNA:* Calculate the binding affinity between the selected 20-nt target sequence and its reverse complementary sequence (target-binding sequence in synthetic sRNA). The predicted hybridization energy should fall within the range of -40 to -20 kcal/mol.

In our original experiments, this parameter was calculated using UNAFold (Markham and Zuker, 2008). However, because this server is currently unavailable, equivalent prediction can be performed using the ViennaRNA package (Varenyk et al., 2023). Thus, the hybridization energy is calculated by submitting both the target-binding sequence and its corresponding mRNA target region to the RNAup web server, included in the ViennaRNA package (http://rna.tbi.univie.ac.at/cgi-bin/RNAWebSuite/RNAup.cgi). In this analysis, the synthetic sRNA target-binding sequence is designated as the query, while the mRNA target region serves as the interaction partner. Default parameters, including a temperature of 37°C and standard energy model settings, are typically applied. RNAup calculates the total binding energy (ΔG) by accounting for both the energetic cost of opening local secondary structures (accessibility) and the free energy released during hybridization between the two sequences. For instance, the hybridization energy of the target-binding sequence of anti-DsRed2 synthetic sRNA and its target mRNA region is -35.66 kcal/mol as shown in Fig. 5A.

When a specific or modulated repression level is required, the binding affinity can be fine-tuned within the effective range by adjusting the length of the target-binding sequence or by introducing mutations within the sequence. Such forward-engineering approaches allow for the rational design of synthetic sRNA variants with predictable and diverse regulatory strengths.

5. *Secondary structure around TIR in mRNA:* Calculate the structural stability of the mRNA TIR. The structural stability plays a pivotal role in determining the accessibility for synthetic sRNA binding. To facilitate efficient hybridization, the predicted Gibbs free energy of the secondary structure within the target mRNA TIR should be higher than -16 kcal/mol. While this thermodynamic parameter was originally calculated through the UNAFold server, due to its unavailability, the RNAfold tool can be used for prediction of TIR secondary structure (http://rna.tbi.univie.ac.at/cgi-bin/RNAWebSuite/RNAfold.cgi). The analysis is performed by inputting the mRNA sequence spanning from 50 nucleotides upstream to 30 nucleotides downstream of the SD sequence, which encompasses the typical ribosome binding region. Specifically, the extracted sequence is processed using the RNAfold web server with default parameters, such as temperature and energy model settings, unless otherwise specified. The predicted minimum free energy structure and its corresponding values are then obtained to ensure that the energetic cost of unfolding pre-existing mRNA structures does not impede effective synthetic sRNA–mRNA base pairing (Fig. 5B).

• Note: Additional computational tools, including RNAstructure (https://rna.urmc.rochester.edu/RNAstructure.html) (Bellaousov et al., 2013) and NUPACK (https://www.nupack.org/) (Fornace et al., 2020), are also suitable for these predictions.

6. *Primer design:* To construct the synthetic sRNA expression vector, we employ an inverse PCR-based site-directed insertion strategy (Fig. S2). The 20-nt target-binding sequence for DsRed2 (indicated in red in Fig. S2A) is partitioned into two approximately equal segments and incorporated as 5' extensions (overhangs) into the forward and reverse primers (Fig. S2B). Specifically, one segment is appended to the 5' end of the forward primer to facilitate its junction with the SgrS scaffold region, while the remaining segment is appended to the 5' end of the reverse primer to anneal with the promoter sequence. The 3' annealing regions of these primers are designed to be complementary to the template vector backbone at the promoter and scaffold junctions, enabling the divergent (back-to-back) amplification of the entire plasmid. Following PCR, the resulting linear amplicons possess terminal sequences that, upon ligation, reconstitute the full 20-nt target-binding sequence. This process yields a functional synthetic sRNA cassette arranged in a promoter–target-binding sequence–SgrS scaffold configuration.

Unlike typical site-directed mutagenesis that often utilizes long, overlapping primers containing the full insertion sequence on both strands, the non-overlapping design employs shorter primers that significantly minimize synthesis costs. This strategy also enhances PCR efficiency by reducing potential self-annealing and primer-dimer formation, making it particularly suitable for the high-throughput construction of various synthetic sRNAs.

• Note: The 3' annealing regions of the primers should be designed to possess a melting temperature (T_m_) within the range of 50 to 55°C. This specific temperature range is recommended to ensure optimal hybridization specificity and efficient PCR process, particularly when amplifying the entire vector backbone.

### B. Plasmid construction

Target-binding sequence can be inserted upstream of the SgrS scaffold by site-directed mutagenesis via inverse PCR. For the anti-DsRed2 synthetic sRNA construct, primers carrying the DsRed2 binding sequence are used to amplify the entire plasmid backbone and introduce the target-binding region in a single PCR step.

1. Prepare PCR reaction (Table 2) using primers (anti-*DsRed2*-F and anti-*DsRed2*-R; Table 1) and pWAS2 plasmid (Fig. S2B).

2. Perform amplification under the cycling conditions described in Table 3 using a high-fidelity DNA polymerase.

3. Resolve PCR product on a 1% agarose gel, and excise the expected band (4.8 kb)

4. Purify the DNA using a commercial gel extraction kit according to the manufacturer’s instructions. Briefly, dissolve the gel slice in solubilization buffer (5× gel volume) at 55°C until completely melted, load onto a silica spin column, wash with ethanol-based wash buffer (750 μl), repeat washing if necessary to remove residual agarose, dry the column, and elute DNA with 30–40 μl elution buffer.

5. Measure DNA concentration using a spectrophotometer (NanoDrop or equivalent).

• Note: Weak, nonspecific, or absent PCR bands may result from primer secondary structures or nonspecific primer annealing. In such cases, amplification specificity and yield can be improved by supplementing the PCR mixture with 1–10% dimethyl sulfoxide or 5–20% glycerol, or by using a higher-fidelity DNA polymerase.

### C. Template removal and plasmid circularization

1. Treat purified PCR product (linearized plasmid with target-binding sequence inserted) with DpnI at 37°C for 1 h (Table 4) to remove methylated template plasmids.

2. Phosphorylate the ends of the PCR product using T4 polynucleotide kinase at 37°C for 1 h (Table 5).

3. Circularize the PCR product using T4 DNA ligase at room temperature for 2 h (Table 6).

4. Store ligated PCR product (circularized plasmid) at 4°C until transformation.

### D. Transformation into *E. coli* DH5α

1. Chemically competent *E. coli* DH5α cells can be prepared using CaCl2 treatment according to the previous procedure (Dagert and Ehrlich, 1979).

2. Add ligation mixtures to competent cells and incubate on ice for 30 min.

3. Perform heat shock at 42°C for 1 min, followed by recovery in LB medium for 1 h at 37°C with shaking at 220 rpm.

4. Spread the transformed cells on LB agar plates supplemented with appropriate antibiotics and incubate overnight.

### E. Colony PCR screening

1. Prepare primers for colony PCR, an insert-specific primer and a backbone primer (Colony-PCR-F/Colony-PCR-R for the anti-DsRed2 synthetic sRNA construct; Table 1).

2. Perform amplification under the thermal cycling conditions described in Tables 7 and 8. Resolve PCR products by agarose gel electrophoresis and identify colonies with a band at 300–350 bp to find correctly constructed plasmids.

### F. Plasmid isolation and sequence verification

1. Incubate colonies, that are positive in colony PCR, in LB containing appropriate antibiotics at 37°C with shaking, and extract plasmid DNA using a commercial miniprep kit according to the manufacturer’s instructions.

2. Verify the sequence of the extracted plasmids by Sanger sequencing using a construct-specific primer (DsRed2 sequencing primer; Table 1) through a commercial sequencing service.

3. Transform the sequence-confirmed plasmid into the appropriate *E. coli* strain for repression experiments.

### G. Validation of synthetic sRNA repression

1. The functional activity of synthetic sRNA can be validated by measuring the repression level of a fluorescent reporter gene.

2. Transform the plasmids encoding the synthetic sRNA, the fluorescent reporter (DsRed2), and the *hfq* variant gene into *E. coli*. As a negative control, prepare cells harboring the reporter plasmid together with a plasmid expressing only the scaffold.

3. Incubate transformed colonies in LB medium overnight at 37°C with shaking supplemented with appropriate antibiotics (100 μg/ml ampicillin for pWAS2 harboring the constructed synthetic sRNA, 50 μg/ml spectinomycin for pSC101-*hfq2*, and 25 μg/ml chloramphenicol for the p15A-based reporter plasmid).

4. Dilute the incubated cells into fresh LB medium containing the same antibiotics and incubate again under identical conditions. After approximately 10 h of growth (stationary phase), collect cells by centrifugation at 13,000 rpm for 1 min. Wash cell pellets once and resuspend in 1 ml of 1× phosphate buffered saline (PBS), followed by a 100-fold dilution in 1× PBS for fluorescence measurements.

5. Measure fluorescence intensity using a flow cytometer (Guava EasyCyte, Millipore, Germany). DsRed2 fluorescence is detected with 561-nm excitation and a 583/26-nm emission filter.

6. Calculate repression efficiency by comparing fluorescence levels between cells expressing synthetic sRNA and scaffold-only control.

• Note: The anti-DsRed2 synthetic sRNA is expected to exhibit approximately 99% repression efficiency, as synthetic sRNAs with a relatively lower binding affinity (e.g., -20 kcal/mol) have previously been shown to yield near-maximal repression levels (Ren et al., 2024).

## Expected Results

Proper implementation of the rational design framework described in this protocol is expected to facilitate the construction of high-efficiency synthetic sRNA systems characterized by robust and predictable repression performance. Application of the established design rules—namely, optimal scaffold selection, precise tuning of target-binding affinity, ensuring TIR accessibility, optimal availability of Hfq protein—is anticipated to significantly enhance synthetic sRNA-mediated translational repression potency.

In the field of metabolic engineering, this framework facilitates the redirection of metabolic fluxes through the rapid and tunable knockdown of target genes. Unlike traditional gene-knockout methods that require laborious chromosomal modifications, synthetic sRNAs enable high-throughput screening by utilizing plasmid-borne regulators that impose minimal metabolic burden on the host compared to protein-based systems like CRISPRi. An advantage of this approach is its capacity to modulate the expression of essential genes, which are indispensable for cellular viability and therefore inaccessible via conventional knockout strategies. Precise modulation of repression levels, achieved through the optimization of synthetic sRNA–mRNA binding affinity, enables the systematic identification of the optimal metabolic balance between biomass accumulation and product synthesis. Furthermore, because the sRNA mechanism is widely conserved, the design rules established here allow for the rapid adaptation of this platform to various bacterial species, significantly accelerating the development of superior microbial factories.

In the context of synthetic biology, the proper application of this framework is expected to regulate complex genetic circuits and maintain circuit stability by mitigating noise-induced activation. For example, by acting as specialized leak suppressors at the post-transcriptional stage, synthetic sRNAs can prevent the spurious activation of synthetic circuits in the absence of activating signals. This integration effectively transforms leaky gene expression into tight, conditional control. In a previous study, synthetic sRNAs enabled positive-feedback circuits—which are typically vulnerable to stochastic activation by intrinsic noise—to remain stable in a non-activated state. Ultimately, the incorporation of synthetic sRNAs as robust and tunable regulatory components significantly enhances the reliability and modularity of complex genetic architectures, providing a foundational platform for the implementation of high-fidelity synthetic biological systems.

## Figures and Tables

**Fig. 1.**
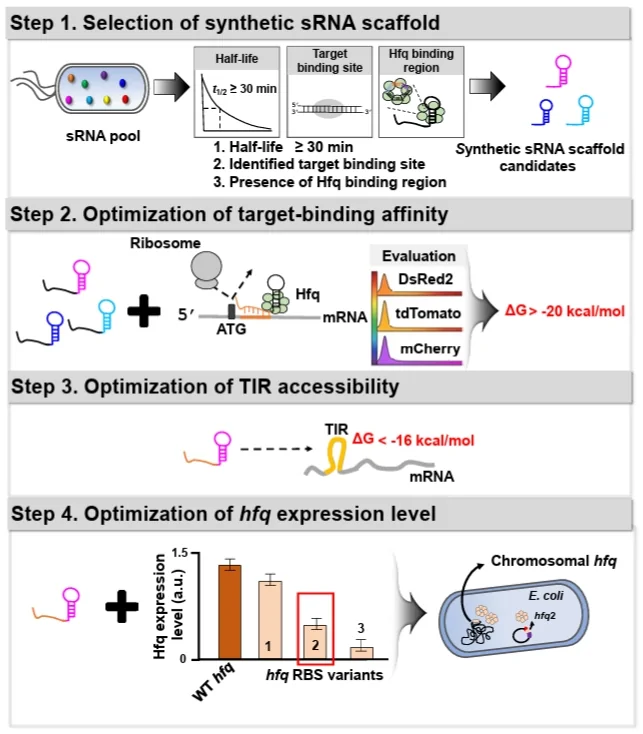
Schematic overview of the rational design workflow and design rules for a high‑efficiency synthetic sRNA regulatory system. The figure illustrates the stepwise design workflow for establishing predictive design rules to modulate gene expression in *E. coli*, which is transferable to other bacterial species. (Step 1) Selection of optimal synthetic sRNA scaffolds from a native sRNA pool based on in vivo half-life and the presence of Hfq-binding motifs. (Step 2) Evaluation of target-binding energy required to ensure robust translational repression. (Step 3) Assessment of the Gibbs free energy of the target mRNA translation initiation region (TIR) to ensure structural accessibility. (Step 4) Fine-tuning of *hfq* expression to achieve maximal gene repression efficiency.

**Fig. 2.**
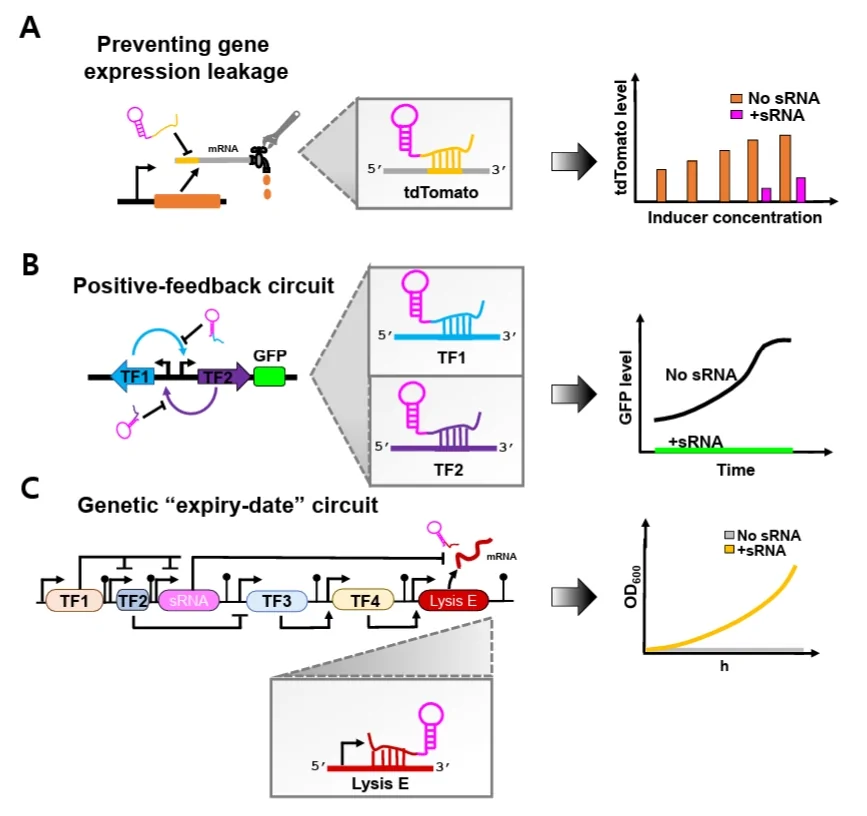
Conceptual overview of synthetic sRNA applications in synthetic biology. (A) Optimized synthetic sRNAs effectively suppress basal gene expression leakage driven by promoters across a wide range of IPTG concentrations, including non-induced conditions. (B) Optimized synthetic sRNAs are incorporated into a positive-feedback circuit to minimize unintended basal activation. (C) Optimized synthetic sRNAs reduce basal leakage of Lysis E expression within the “expiry-date” circuit, restoring normal cellular growth under permissive conditions.

**Fig. 3.**
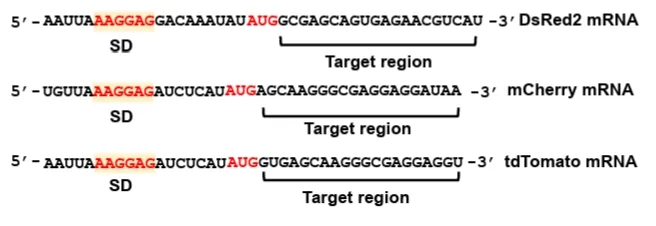
Genetic sequences of the SD sequences and target regions in fluorescent reporter genes. The Shine-Dalgarno (SD) sequences and corresponding target regions of three fluorescent genes—*DsRed2*, *mCherry*, and *tdTomato*—are shown. These mRNA target regions are specifically recognized by complementary sequences (target-binding sequences) within synthetic sRNAs.

**Fig. 4.**
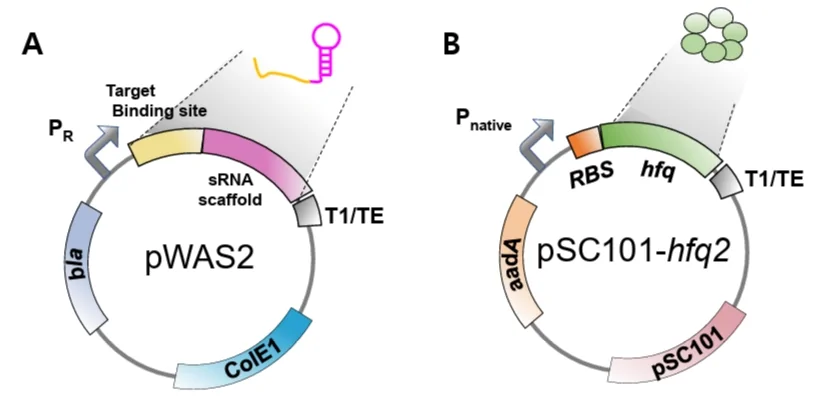
Genetic structures of the plasmids. (A) Schematic structure of the pWAS2 plasmid for synthetic sRNA expression. The plasmid harbors the ColE1 replication origin, the *bla* gene conferring ampicillin resistance, and a synthetic sRNA cassette consisting of a target-binding sequence and an sRNA scaffold under the control of the phage λ P_R_ promoter. (B) Schematic structure of the pSC101-*hfq2* plasmid for additional *hfq* expression. Hfq expression level is modulated by modifying its RBS (Fig. S1). The plasmid contains the pSC101 replication origin, the *aadA* gene conferring spectinomycin resistance, and the *hfq* gene with modified RBS under its native promoter.

**Fig. 5.**
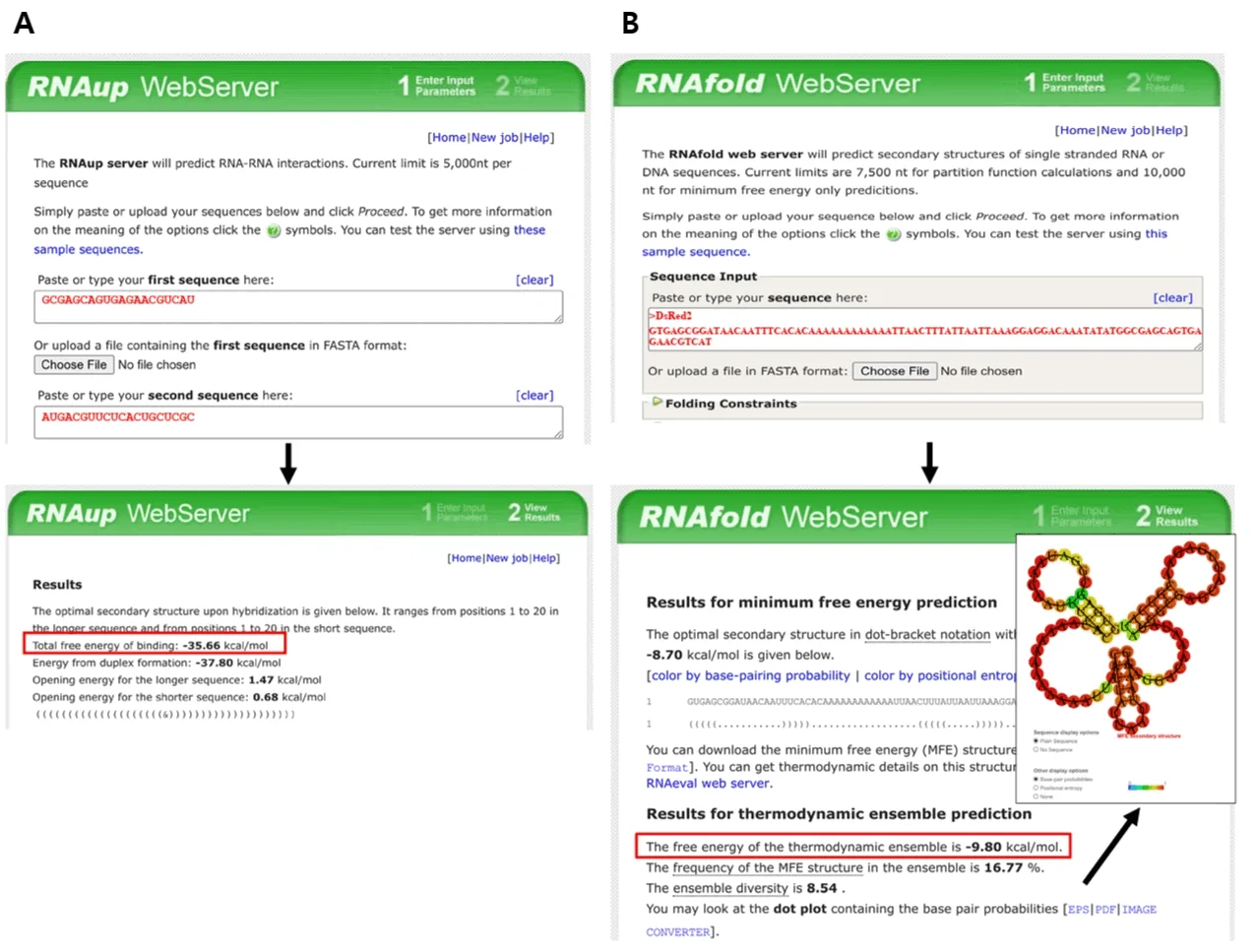
Free-energy prediction using ViennaRNA tools. (A) Hybridization energy between the target-binding sequence of anti-DsRed2 synthetic sRNA and its reverse complement (target region in DsRed2 mRNA) is calculated using RNAup. (B) The sequence spanning from 50 nucleotides upstream of the SD sequence to 30 nucleotides downstream is analyzed using RNAfold to evaluate TIR secondary structure. Red boxes indicate predicted free-energy values.

**Table 1.** Oligonucleotides used in this protocol

Constructs	Oligo name	Oligonucleotides (5’-3’)
pWAS2	anti-DsRed2-F	CAcTGCTCGCATCACCCGCCAGCAGATTA
	anti-DsRed2-R	CTCACGTCATGCAACCATTATCACCGCCA
	Colony-PCR-F	GGCGGTGATAATGGTTGCATGACGTTCTCA
	Colony-PCR-R	CTTATCGCATGCCTAGGTAAACCCAGGAGG
	Sequencing	CTAGGTAAACCCAGGAGG

**Table 2.** Composition of the site-directed mutagenesis PCR master mixture

Component	Amount per reaction (μl)	Final amount
Q5 High-Fidelity DNA polymerase (2 U/μl)	1	1 U
5× reaction buffer	5	1×
dNTP mix (25 mM of each dNTP)	0.5	0.25 mM
DNA template	1	100 ng
Forward primer (10 μM)	1	0.2 μM
Reverse primer (10 μM)	1	0.2 μM
Distilled water	15.5	
Total reaction volume	25	

**Table 3.** Thermal cycling conditions for site-directed mutagenesis PCR

Step	Temperature	Duration	Cycles
**Initial denaturation**	98°C	30 s	1
**Denaturation**	98°C	5–10 s	25–35
**Annealing**	50–72°C	10–30 s	
**Extension**	72°C	20–30 s/kb	
**Final extension**	72°C	5 min	1
**Hold**	8°C	∞	-

**Table 4.** Composition for the DpnI digestion

Component	Amount per sample (μl)	Final
DpnI restriction enzyme (20 U/μl)	1	20 U
10× reaction buffer	2	１×
Gel-extracted PCR product	17	
Total reaction volume	20	

**Table 5.** Composition for DNA phosphorylation

Component	Amount per sample (μl)	Final
T4 polynucleotide kinase (20 U/μl)	1	20 U
10× T4 Polynucleotide kinase reaction buffer	2	1×
DpnI-digested PCR product	17	
Total reaction volume	20	

**Table 6.** Composition for the T4 DNA ligation

Component	Amount per sample (μl)	Final
Phosphorylated PCR product	17	
T4 DNA ligase (10 U/μl)	1	10 U
10× T4 DNA ligase buffer	2	1×
Total reaction volume	20	

**Table 7.** Composition of the colony PCR master mix

Component	Amount per reaction (μl)	Final
Taq DNA polymerase (2 U/μl)	0.5	1 U
10× reaction buffer	2	1×
dNTP mix (10 mM of each dNTP)	1	0.2 mM
DNA template	1	5–10 ng
Forward primer (10 μM)	1	0.2 μM
Reverse primer (10 μM)	1	0.2 μM
Distilled water	13.5	
Total reaction volume	20	

Note: For efficient plasmid verification or fragment amplification, one primer should anneal to the vector backbone and the other to the insert region.

**Table 8.** Thermal cycling conditions for colony PCR

Step	Temperature	Duration	Cycles
**Initial denaturation**	95°C	2 min	1
**Denaturation**	95°C	20 s	25–40
**Annealing**	50–72°C	30 s	
**Extension**	72°C	1 min/kb	
**Final extension**	72°C	5 min	1
**Hold**	8°C	∞	-

## Supplementary Information

Fig. S1. Genetic structure of *hfq2* variant. (A) Sequence modification introduced into the RBS of the *hfq* gene while preserving the native *hfq* Shine-Dalgarno (SD) sequence and coding sequence (CDS). Blue nucleotides represent the native SD sequence (AAGGAA), green nucleotides indicate introduced nucleotide changes in the RBS, and red nucleotides correspond to the N-terminal region of the *hfq* CDS. (B) Complete nucleotide sequence of the *hfq2* variant construct spanning from the native promoter to transcription terminators. The promoter sequence is shown in orange, the UTR region in green, and the translation start codon (ATG) in red, the *hfq* CDS in underlined, and the transcription terminators (T1/TE) in blue.

Fig. S2. Construction of pWAS2 plasmid expressing an SgrS-based synthetic sRNA targeting DsRed2. (A) Sequence context of the *DsRed2* target region. The SD sequence is shown in blue, the start codon (ATG) in green, and the region targeted by the anti-DsRed2 synthetic sRNA in red. (B) Primers used for constructing the anti-DsRed2 synthetic sRNA by site-directed mutagenesis PCR. The primers, anti-DsRed2-F and anti-DsRed2-R (yellow background), introduce a 20-bp target-binding sequence colored in red (reverse complementary to the *DsRed2* mRNA). The annealing sequence to SgrS scaffold is colored in green and that to the *P*_R_ promoter is colored in black.
