# Supplementary material for: Design guide for synthetic small regulatory RNAs for high-efficiency gene knockdown in bacteria
Source: J Microbiol. 2026 Jul 6;64(7):e2603026. doi: 10.71150/jm.2603026 (PMC13712874; doi:10.71150/jm.2603026)
Supplement: Supplementary file 1 [file jm-2603026-Supplementary-Fig-S1.pdf]

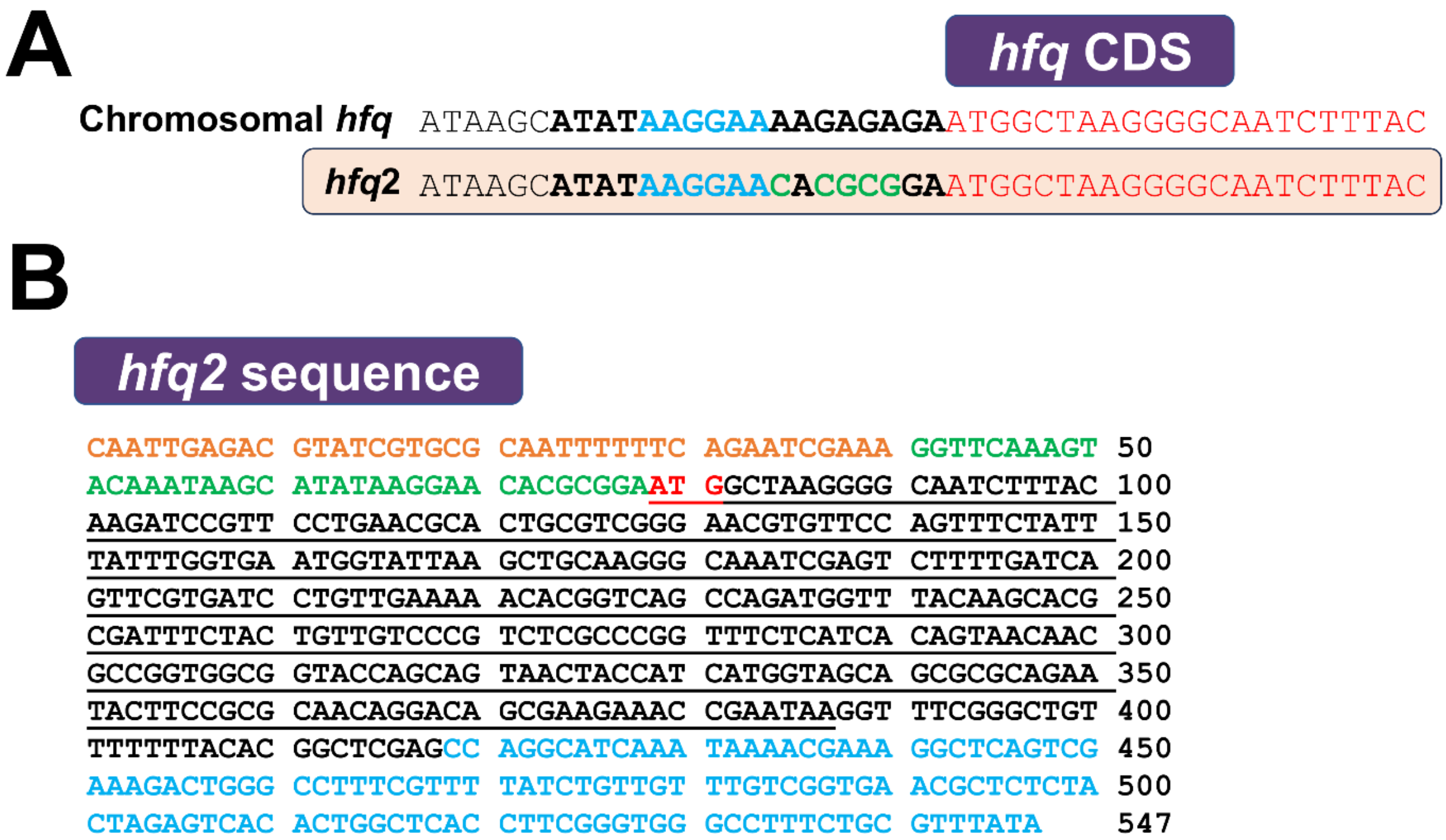

Fig. S1. Genetic structure of *hfq*2 variant. (A) Sequence modification introduced into the RBS of the *hfq* gene while preserving the native *hfq* Shine-Dalgarno (SD) sequence and coding sequence (CDS). Blue nucleotides represent the native SD sequence (AAGGAA), green nucleotides indicate introduced nucleotide changes in the RBS, and red nucleotides correspond to the N-terminal region of the *hfq* CDS. (B) Complete nucleotide sequence of the *hfq2* variant construct spanning from the native promoter to transcription terminators. The promoter sequence is shown in orange, the UTR region in green, and the translation start codon (ATG) in red, the *hfq* CDS in underlined, and the transcription terminators (T1/TE) in blue.
