# Supplementary material for: Design guide for synthetic small regulatory RNAs for high-efficiency gene knockdown in bacteria
Source: J Microbiol. 2026 Jul 6;64(7):e2603026. doi: 10.71150/jm.2603026 (PMC13712874; doi:10.71150/jm.2603026)
Supplement: Supplementary file 2 [file jm-2603026-Supplementary-Fig-S2.pdf]

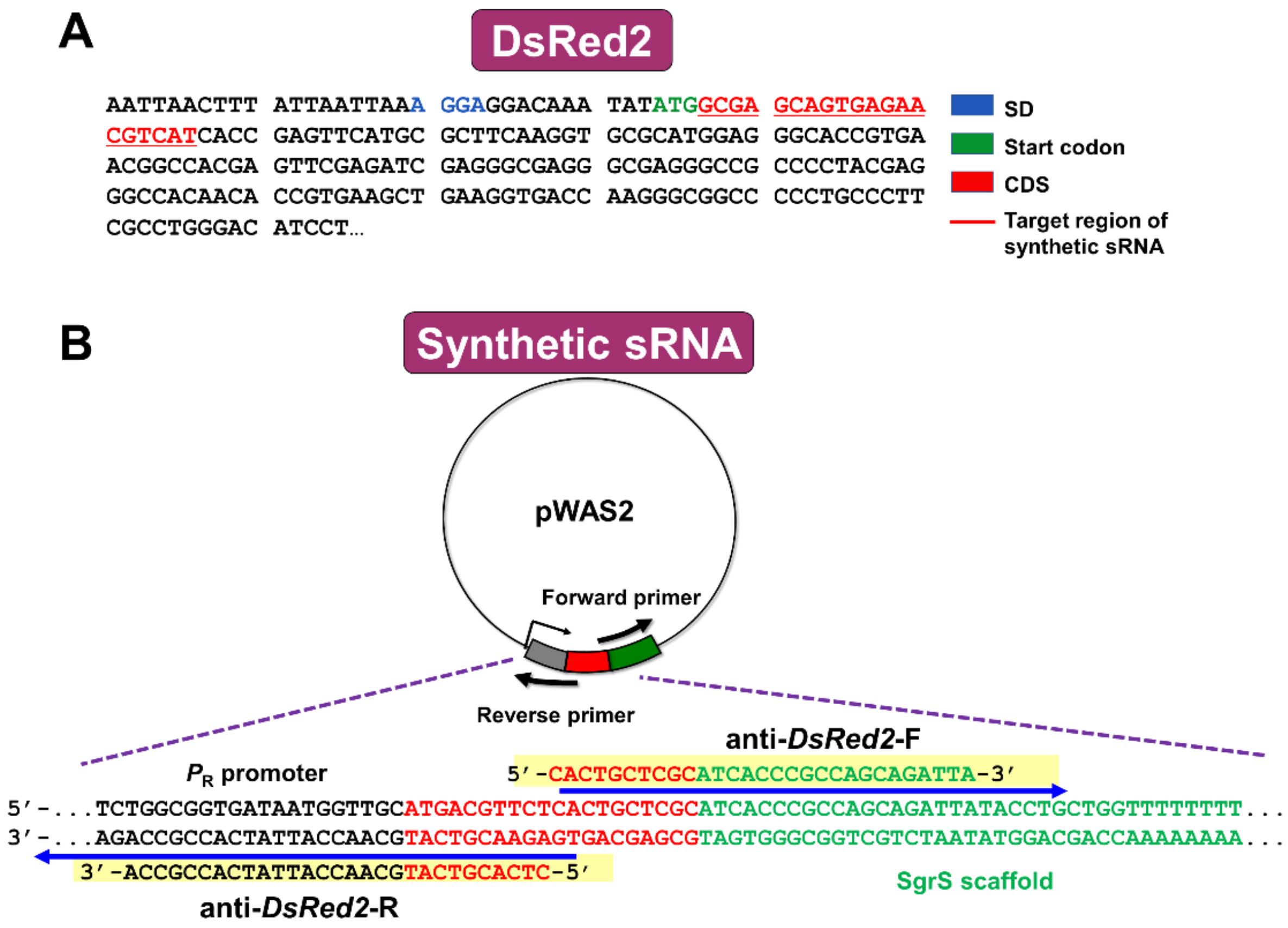

Fig. S2. Construction of pWAS2 plasmid expressing an SgrS-based synthetic sRNA targeting DsRed2. (A) Sequence context of the *DsRed2* target region. The SD sequence is shown in blue, the start codon (ATG) in green, and the region targeted by the anti-DsRed2 synthetic sRNA in red. (B) Primers used for constructing the anti-DsRed2 synthetic sRNA by site-directed mutagenesis PCR. The primers, anti-DsRed2-F and anti-DsRed2-R (yellow background), introduce a 20-bp target-binding sequence colored in red (reverse complementary to the *DsRed2* mRNA). The annealing sequence to SgrS scaffold is colored in green and that to the P_R promoter is colored in black.
